# Supplementary material for: HIVprotI: an integrated web based platform for prediction and design of HIV proteins inhibitors
Source: J Cheminform. 2018 Mar 9;10:12. doi: 10.1186/s13321-018-0266-y (PMC5845081; doi:10.1186/s13321-018-0266-y)
Supplement: Supplementary file 2 — Additional file 2. Source code of HIVProtI web server. [file 13321_2018_266_MOESM2_ESM.zip › HIVprotI_Source-code/hivprotI/weka-3-6-6/doc/weka/gui/visualize/AttributePanel.html]

AttributePanel


---


|  |  |  |
| --- | --- | --- |
| **Overview** \| **Package** \| **Class** \| **Tree** \| **Deprecated** \| **Index** \| **Help** \| **Weka's home** | |  |
| PREV CLASS   **NEXT CLASS** | **FRAMES**    **NO FRAMES**     **All Classes** |  |
| SUMMARY: NESTED \| FIELD \| CONSTR \| METHOD | DETAIL: FIELD \| CONSTR \| METHOD |  |


---


## weka.gui.visualize Class AttributePanel

```
java.lang.Object
  java.awt.Component
      java.awt.Container
          javax.swing.JComponent
              javax.swing.JScrollPane
                  weka.gui.visualize.AttributePanel
```

**All Implemented Interfaces:**: java.awt.image.ImageObserver, java.awt.MenuContainer, java.io.Serializable, javax.accessibility.Accessible, javax.swing.ScrollPaneConstants

---

``` public class AttributePanel extends javax.swing.JScrollPane ```

This panel displays one dimensional views of the attributes in a
dataset. Colouring is done on the basis of a column in the dataset or
an auxiliary array (useful for colouring cluster predictions).

**Version:**
:   $Revision: 7059 $

**Author:**
:   Malcolm Ware (mfw4@cs.waikato.ac.nz), Mark Hall (mhall@cs.waikato.ac.nz)

**See Also:**: Serialized Form

---

**Nested Class Summary**

**Nested classes/interfaces inherited from class javax.swing.JComponent**

`javax.swing.JComponent.AccessibleJComponent`

**Nested classes/interfaces inherited from class java.awt.Component**

`java.awt.Component.BaselineResizeBehavior`


**Field Summary**

**Fields inherited from class javax.swing.JComponent**

`TOOL_TIP_TEXT_KEY, UNDEFINED_CONDITION, WHEN_ANCESTOR_OF_FOCUSED_COMPONENT, WHEN_FOCUSED, WHEN_IN_FOCUSED_WINDOW`

**Fields inherited from class java.awt.Component**

`BOTTOM_ALIGNMENT, CENTER_ALIGNMENT, LEFT_ALIGNMENT, RIGHT_ALIGNMENT, TOP_ALIGNMENT`

**Fields inherited from interface javax.swing.ScrollPaneConstants**

`COLUMN_HEADER, HORIZONTAL_SCROLLBAR, HORIZONTAL_SCROLLBAR_ALWAYS, HORIZONTAL_SCROLLBAR_AS_NEEDED, HORIZONTAL_SCROLLBAR_NEVER, HORIZONTAL_SCROLLBAR_POLICY, LOWER_LEADING_CORNER, LOWER_LEFT_CORNER, LOWER_RIGHT_CORNER, LOWER_TRAILING_CORNER, ROW_HEADER, UPPER_LEADING_CORNER, UPPER_LEFT_CORNER, UPPER_RIGHT_CORNER, UPPER_TRAILING_CORNER, VERTICAL_SCROLLBAR, VERTICAL_SCROLLBAR_ALWAYS, VERTICAL_SCROLLBAR_AS_NEEDED, VERTICAL_SCROLLBAR_NEVER, VERTICAL_SCROLLBAR_POLICY, VIEWPORT`

**Fields inherited from interface java.awt.image.ImageObserver**

`ABORT, ALLBITS, ERROR, FRAMEBITS, HEIGHT, PROPERTIES, SOMEBITS, WIDTH`


**Constructor Summary**

`AttributePanel()`

`AttributePanel(java.awt.Color background)`
  
          This constructs an attributePanel.


| **Method Summary** | |
| --- | --- |
| `void` | `addAttributePanelListener(AttributePanelListener a)`             Add a listener to the list of things listening to this panel |
| `static void` | `main(java.lang.String[] args)`             Main method for testing this class. |
| `void` | `setCindex(int c)`             Set the index of the attribute by which to colour the data. |
| `void` | `setCindex(int c, double h, double l)`             Set the index of the attribute by which to colour the data. |
| `void` | `setColours(FastVector cols)`             Sets a list of colours to use for colouring data points |
| `void` | `setInstances(Instances ins)`             This sets the instances to be drawn into the attribute panel |
| `void` | `setX(int x)`             shows which bar is the current x attribute. |
| `void` | `setY(int y)`             shows which bar is the current y attribute. |

**Methods inherited from class javax.swing.JScrollPane**

`createHorizontalScrollBar, createVerticalScrollBar, getAccessibleContext, getColumnHeader, getCorner, getHorizontalScrollBar, getHorizontalScrollBarPolicy, getRowHeader, getUI, getUIClassID, getVerticalScrollBar, getVerticalScrollBarPolicy, getViewport, getViewportBorder, getViewportBorderBounds, isValidateRoot, isWheelScrollingEnabled, setColumnHeader, setColumnHeaderView, setComponentOrientation, setCorner, setHorizontalScrollBar, setHorizontalScrollBarPolicy, setLayout, setRowHeader, setRowHeaderView, setUI, setVerticalScrollBar, setVerticalScrollBarPolicy, setViewport, setViewportBorder, setViewportView, setWheelScrollingEnabled, updateUI`

**Methods inherited from class javax.swing.JComponent**

`addAncestorListener, addNotify, addVetoableChangeListener, computeVisibleRect, contains, createToolTip, disable, enable, firePropertyChange, firePropertyChange, firePropertyChange, getActionForKeyStroke, getActionMap, getAlignmentX, getAlignmentY, getAncestorListeners, getAutoscrolls, getBaseline, getBaselineResizeBehavior, getBorder, getBounds, getClientProperty, getComponentPopupMenu, getConditionForKeyStroke, getDebugGraphicsOptions, getDefaultLocale, getFontMetrics, getGraphics, getHeight, getInheritsPopupMenu, getInputMap, getInputMap, getInputVerifier, getInsets, getInsets, getListeners, getLocation, getMaximumSize, getMinimumSize, getNextFocusableComponent, getPopupLocation, getPreferredSize, getRegisteredKeyStrokes, getRootPane, getSize, getToolTipLocation, getToolTipText, getToolTipText, getTopLevelAncestor, getTransferHandler, getVerifyInputWhenFocusTarget, getVetoableChangeListeners, getVisibleRect, getWidth, getX, getY, grabFocus, isDoubleBuffered, isLightweightComponent, isManagingFocus, isOpaque, isOptimizedDrawingEnabled, isPaintingForPrint, isPaintingTile, isRequestFocusEnabled, paint, paintImmediately, paintImmediately, print, printAll, putClientProperty, registerKeyboardAction, registerKeyboardAction, removeAncestorListener, removeNotify, removeVetoableChangeListener, repaint, repaint, requestDefaultFocus, requestFocus, requestFocus, requestFocusInWindow, resetKeyboardActions, reshape, revalidate, scrollRectToVisible, setActionMap, setAlignmentX, setAlignmentY, setAutoscrolls, setBackground, setBorder, setComponentPopupMenu, setDebugGraphicsOptions, setDefaultLocale, setDoubleBuffered, setEnabled, setFocusTraversalKeys, setFont, setForeground, setInheritsPopupMenu, setInputMap, setInputVerifier, setMaximumSize, setMinimumSize, setNextFocusableComponent, setOpaque, setPreferredSize, setRequestFocusEnabled, setToolTipText, setTransferHandler, setVerifyInputWhenFocusTarget, setVisible, unregisterKeyboardAction, update`

**Methods inherited from class java.awt.Container**

`add, add, add, add, add, addContainerListener, addPropertyChangeListener, addPropertyChangeListener, applyComponentOrientation, areFocusTraversalKeysSet, countComponents, deliverEvent, doLayout, findComponentAt, findComponentAt, getComponent, getComponentAt, getComponentAt, getComponentCount, getComponents, getComponentZOrder, getContainerListeners, getFocusTraversalKeys, getFocusTraversalPolicy, getLayout, getMousePosition, insets, invalidate, isAncestorOf, isFocusCycleRoot, isFocusCycleRoot, isFocusTraversalPolicyProvider, isFocusTraversalPolicySet, layout, list, list, locate, minimumSize, paintComponents, preferredSize, printComponents, remove, remove, removeAll, removeContainerListener, setComponentZOrder, setFocusCycleRoot, setFocusTraversalPolicy, setFocusTraversalPolicyProvider, transferFocusBackward, transferFocusDownCycle, validate`

**Methods inherited from class java.awt.Component**

`action, add, addComponentListener, addFocusListener, addHierarchyBoundsListener, addHierarchyListener, addInputMethodListener, addKeyListener, addMouseListener, addMouseMotionListener, addMouseWheelListener, bounds, checkImage, checkImage, contains, createImage, createImage, createVolatileImage, createVolatileImage, dispatchEvent, enable, enableInputMethods, firePropertyChange, firePropertyChange, firePropertyChange, firePropertyChange, firePropertyChange, getBackground, getBounds, getColorModel, getComponentListeners, getComponentOrientation, getCursor, getDropTarget, getFocusCycleRootAncestor, getFocusListeners, getFocusTraversalKeysEnabled, getFont, getForeground, getGraphicsConfiguration, getHierarchyBoundsListeners, getHierarchyListeners, getIgnoreRepaint, getInputContext, getInputMethodListeners, getInputMethodRequests, getKeyListeners, getLocale, getLocation, getLocationOnScreen, getMouseListeners, getMouseMotionListeners, getMousePosition, getMouseWheelListeners, getName, getParent, getPeer, getPropertyChangeListeners, getPropertyChangeListeners, getSize, getToolkit, getTreeLock, gotFocus, handleEvent, hasFocus, hide, imageUpdate, inside, isBackgroundSet, isCursorSet, isDisplayable, isEnabled, isFocusable, isFocusOwner, isFocusTraversable, isFontSet, isForegroundSet, isLightweight, isMaximumSizeSet, isMinimumSizeSet, isPreferredSizeSet, isShowing, isValid, isVisible, keyDown, keyUp, list, list, list, location, lostFocus, mouseDown, mouseDrag, mouseEnter, mouseExit, mouseMove, mouseUp, move, nextFocus, paintAll, postEvent, prepareImage, prepareImage, remove, removeComponentListener, removeFocusListener, removeHierarchyBoundsListener, removeHierarchyListener, removeInputMethodListener, removeKeyListener, removeMouseListener, removeMouseMotionListener, removeMouseWheelListener, removePropertyChangeListener, removePropertyChangeListener, repaint, repaint, repaint, resize, resize, setBounds, setBounds, setCursor, setDropTarget, setFocusable, setFocusTraversalKeysEnabled, setIgnoreRepaint, setLocale, setLocation, setLocation, setName, setSize, setSize, show, show, size, toString, transferFocus, transferFocusUpCycle`

**Methods inherited from class java.lang.Object**

`equals, getClass, hashCode, notify, notifyAll, wait, wait, wait`

**Constructor Detail**

### AttributePanel

```
public AttributePanel()
```

---


### AttributePanel

```
public AttributePanel(java.awt.Color background)
```

:   This constructs an attributePanel.


**Method Detail**

### addAttributePanelListener

```
public void addAttributePanelListener(AttributePanelListener a)
```

:   Add a listener to the list of things listening to this panel

    :   **Parameters:**: `a` - the listener to notify when attribute bars are clicked on

---


### setCindex

```
public void setCindex(int c,
                      double h,
                      double l)
```

:   Set the index of the attribute by which to colour the data. Updates
    the number of entries in the colour list if there are more values
    for this new attribute than previous ones.

    :   **Parameters:**: `c` - the index of the attribute to colour on: `h` - maximum value of this attribute: `l` - minimum value of this attribute

---


### setCindex

```
public void setCindex(int c)
```

:   Set the index of the attribute by which to colour the data. Updates
    the number of entries in the colour list if there are more values
    for this new attribute than previous ones.

    :   **Parameters:**: `c` - the index of the attribute to colour on

---


### setColours

```
public void setColours(FastVector cols)
```

:   Sets a list of colours to use for colouring data points

    :   **Parameters:**: `cols` - a list of java.awt.Color

---


### setInstances

```
public void setInstances(Instances ins)
                  throws java.lang.Exception
```

:   This sets the instances to be drawn into the attribute panel

    :   **Parameters:**: `ins` - The instances. **Throws:**: `java.lang.Exception`

---


### setX

```
public void setX(int x)
```

:   shows which bar is the current x attribute.

    :   **Parameters:**: `x` - The attributes index.

---


### setY

```
public void setY(int y)
```

:   shows which bar is the current y attribute.

    :   **Parameters:**: `y` - The attributes index.

---


### main

```
public static void main(java.lang.String[] args)
```

:   Main method for testing this class.

    :   **Parameters:**: `args` - first argument should be an arff file. Second argument can be an optional class col


---


|  |  |  |
| --- | --- | --- |
| **Overview** \| **Package** \| **Class** \| **Tree** \| **Deprecated** \| **Index** \| **Help** \| **Weka's home** | |  |
| PREV CLASS   **NEXT CLASS** | **FRAMES**    **NO FRAMES**     **All Classes** |  |
| SUMMARY: NESTED \| FIELD \| CONSTR \| METHOD | DETAIL: FIELD \| CONSTR \| METHOD |  |


---
